# Dielectric-Like Behavior of Graphene in Au Plasmon Resonator

**DOI:** 10.1186/s11671-016-1753-6

**Published:** 2016-12-07

**Authors:** Junku Liu, Qunqing Li, Mo Chen, Mengxin Ren, Lihui Zhang, Lin Xiao, Kaili Jiang, Shoushan Fan

**Affiliations:** 1 Nanophotonics and Optoelectronics Research Center, Qian Xuesen Laboratory of Space Technology, China Academy of Space Technology, Beijing, 100094 China; 2 State Key Laboratory of Low-Dimensional Quantum Physics, Department of Physics and Tsinghua-Foxconn Nanotechnology Research Center, Tsinghua University, Beijing, 100084 China; 3 The Key Laboratory of Weak-Light Nonlinear Photonics, Ministry of Education, School of Physics and TEDA Applied Physics School, Nankai University, Tianjin, 300457 China

**Keywords:** Graphene, Plasmon resonator, SiO_2_ film, Dielectric-like behavior

## Abstract

Graphene has proven to be a promising conductive layer in fabricating optical plasmon resonators on insulator substrate using electron beam lithography and has the potential to construct electrically controlled active plasmon resonators. In this study, we investigate the effect of graphene on plasmon resonance using graphene and Au plasmon resonator system as a model at visible and near-infrared wavelength. Our experiment data show that the presence of graphene does not weaken and annihilate the plasmon resonance peaks, instead it predominantly makes the peaks redshift, which is similar to the behavior of depositing SiO_2_ film on Au plasmon resonators. This fact indicates that graphene predominantly exhibits dielectric-like behavior at visible and near-infrared wavelength, which can be attributed to the low carrier density in graphene compared with metals.

**Electronic supplementary material:**

The online version of this article (doi:10.1186/s11671-016-1753-6) contains supplementary material, which is available to authorized users.

## Background

Optical plasmon resonators are artificial nanostructures on sub-wavelength scale, with the ability to engineer electromagnetic space and control the propagation of electromagnetic wave [1, 2]. In the past few years, important breakthroughs have been realized using optical plasmon resonators, such as negative refraction [3, 4], superlens [5, 6], and induced transparency [7]. These achievements inspire researchers to design and fabricate more optical nanostructures with unique properties in order to explore novel optical phenomena and construct active optical plasmon resonators [8–14], while fabrication of optical plasmon resonators on sub-nanometer scales on insulator substrate is still a challenge due to the over accumulation of electrons on the substrate during electron beam lithography (EBL), which deflects the beam and distorts the pattern [15].

Graphene, a two-dimensional plane of carbon atoms arranged in a honeycomb lattice, is a semimetal with zero energy band gap. A single-layer graphene has a transmission of 97.7% at visible and near-infrared region [16] and has been used as a promising transparent conductor in touch screens, flexible displays, printable electronics, solid-state lighting, and thin-film photovoltaics [17]. Recently, graphene has also been proven to be a promising conductive layer in fabricating optical plasmon resonators on insulator substrate using EBL. Compared with conventional metals, using graphene as discharge layer can make smaller, more accurate, and more complex optical plasmon resonators. What is more, the plasmon resonators with graphene still exhibit clear plasmon resonance peaks [18]. Therefore, it is necessary to investigate the effect of graphene on optical plasmon resonators.

In addition to be a promising conductive layer in fabricating complex optical plasmon resonators, graphene also has the potential to construct electrically controlled active plasmon resonators at broadband wavelength [11–14, 19, 20] by tuning carrier density in graphene. Carrier density in graphene can be increased up to 10^14^/cm^2^ using highly efficient ion-gel dielectric [21], which is still much lower than that in metals. Therefore, plasmonics in graphene is usually used in mid-infrared frequency [22]. At visible and near-infrared (NIR) frequencies, the hybrid graphene-plasmon resonator system has attracted wide attention in constructing electrically tunable optical plasmon resonators [11, 14, 20]. These applications all have made it necessary to understand the effect of graphene on the optical plasmon resonators in the hybrid system.

In this work, we investigated the effect of graphene on plasmon resonator system at visible and near-infrared wavelength using Au plasmon resonators as a model system. The experiment data show that the plasmon resonance peaks exhibit clear redshift no matter whether graphene is placed below or above the Au plasmon resonators, and the plasmon resonance peaks’ shape experience little change. Frequency redshift also appears when a SiO_2_ film is deposited on the Au plasmon resonators. Since depositing SiO_2_ film or graphene on the Au plasmon resonators both exhibit the same effect, this indicates that graphene predominantly shows dielectric-like behavior at visible and NIR frequencies. The research is beneficial for understanding the role of graphene in Au plasmon resonators at visible and NIR frequency.

## Results and Discussion

**Fig. 1:**
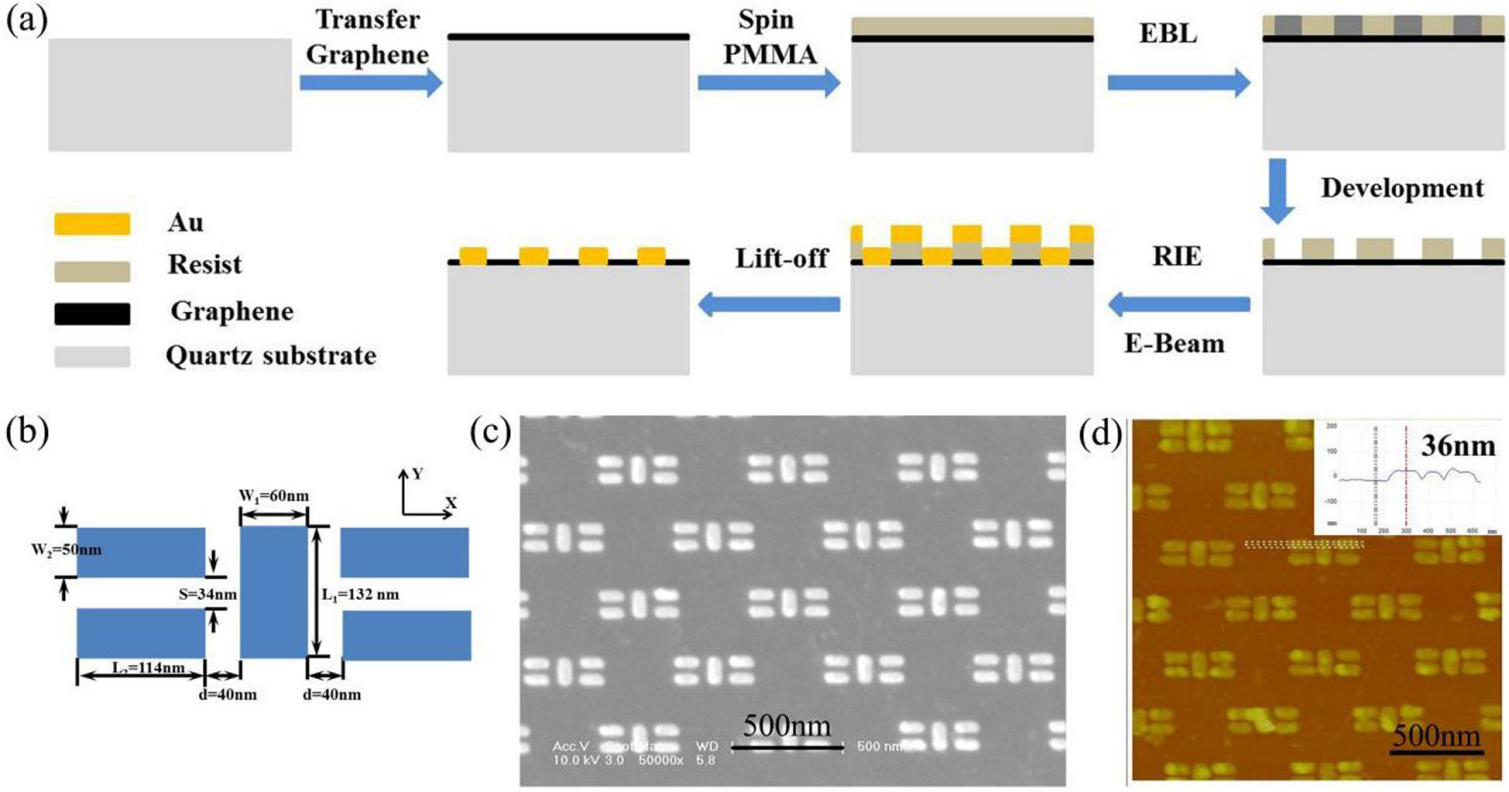
**a** Schematic illustration of the process of fabricating Au plasmon resonator array on quartz substrate using graphene as discharge layer. **b** Schematic diagram of an Au plasmon resonator design. **c** SEM image of the fabricated Au plasmon resonator array. **d** AFM image of the fabricated Au plasmon resonator array; the *inset* in (**d**) shows the height profile of the fabricated Au plasmon resonator array

Figure 1a shows the fabrication process of Au plasmon resonator array on quartz substrate using graphene as discharge layer. CVD graphene was first transferred to quartz substrate, then the quartz substrate coated with graphene was spin coated with resist PMMA 950K A2. After EBL exposure and development, 1 nm Cr and 30 nm Au were deposited. We obtained Au plasmon resonator array after lift-off using acetone at 40 °C. Figure 1b shows the design of an Au plasmon resonator array. The Au plasmon resonator array is characterized by optical anisotropy in terms of X-polarized and Y-polarized excitation with multiple resonance peaks at visible and NIR wavelength [23]. All these features provide great convenience in investigating the effect of graphene on optical plasmon resonators at visible and NIR frequency. In the EBL process, the length L_*i*_ and width W_*i*_ (*i* = 1,2) were all decreased by 16 nm in order to offset the increase caused by RIE. Figure 1c, d show the SEM and AFM images of the fabricated Au plasmon resonator array on quartz substrate, respectively. These images indicate that the nanostructures with the critical dimension of 34 nm can be realized using graphene as discharge layer on quartz substrate. What is more, the corner of the nanostructure is more sharply defined on graphene than that on 10 nm Cr films [18]. The inset in Fig. 1d shows the height profile of the fabricated Au plasmon resonators is about 36 nm, which is consistent with e-beam deposition thickness.

**Fig. 2:**
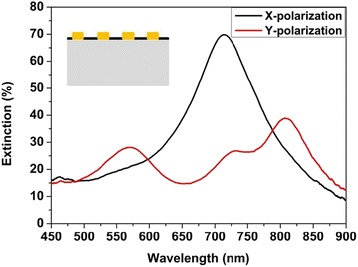
Experimental extinction spectra of Au plasmon resonator array on quartz substrate with graphene residue excited by normal incident X- and Y-polarized beams. The *inset* shows the schematic illustration of the measured system consisting of Au plasmon resonators and graphene

Figure 2 shows the experimental extinction spectra of the Au plasmon resonator array on quartz substrate with graphene residue (Additional file 1: Figure S1) excited by normal incident X- and Y-polarized beams. The sample footprint is 30 × 30 μm^2^, and the spectra are collected from 20 × 20 μm^2^ in the sample center using a ×10 objective. The sample exhibits one absorption peak at about 715 nm under X-polarized light excitation (see black curve), but two plasmon resonance peaks occur at around 570 and 807 nm for Y-polarized light, respectively (see red curve). All of these absorption peaks are consistent with the numerical simulation results when only Au plasmon resonator array on quartz [23] is considered. It indicates that the presence of graphene (Additional file 2: Figure S2) does not weaken and annihilate the Au plasmon resonance peaks, but the effect of graphene in the Au plasmon resonators is not clear.

**Fig. 3:**
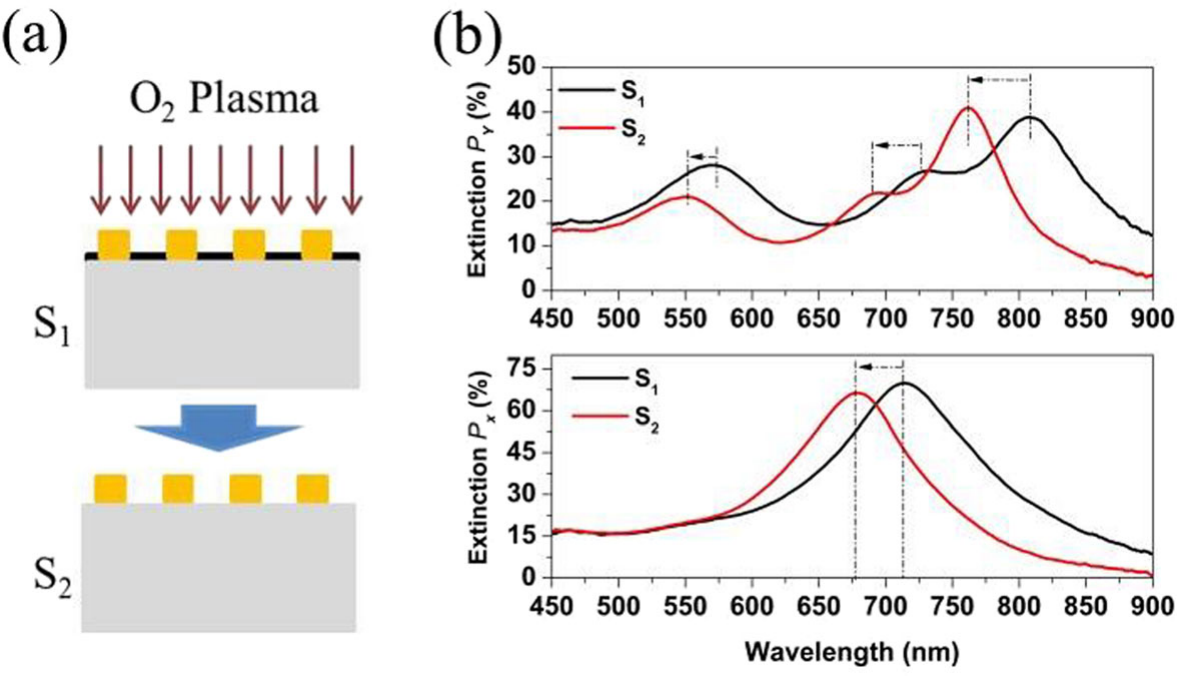
**a** Schematic illustration of removing graphene by using O_2_ RIE. **b** The extinction spectra of the Au plasmon resonator array with and without graphene excited by X- and Y-polarized light at normal incidence

In order to reveal the effect of graphene on the optical spectra of the Au plasmon resonator array at visible and NIR wavelength, we compared the extinction spectra of sample with and without graphene. Graphene in the fabricated Au plasmon resonator array was removed using O_2_ RIE. The RIE condition was appropriate to removing graphene while having little effect on Au nanostructure. Figure 3a shows a schematic illustration of the sample with and without graphene. The black lines in the two panels of Fig. 3b show the extinction spectra of the Au plasmon resonator array with graphene excited by Y- and X-polarized light at normal incidence, respectively. After graphene was removed, the extinction spectra excited by Y- and X-polarized light at normal incidence were showed as red line in Fig. 3b. The data show that all of the plasmon resonance peaks excited by X- and Y-polarized light exhibit a clear blue-shift after removing graphene. The absorption of graphene is only 2.3% per layer at visible and NIR wavelength, which makes a little contribution to broaden the full width at half maximum (FWHM) of plasmon resonance.

**Fig. 4:**
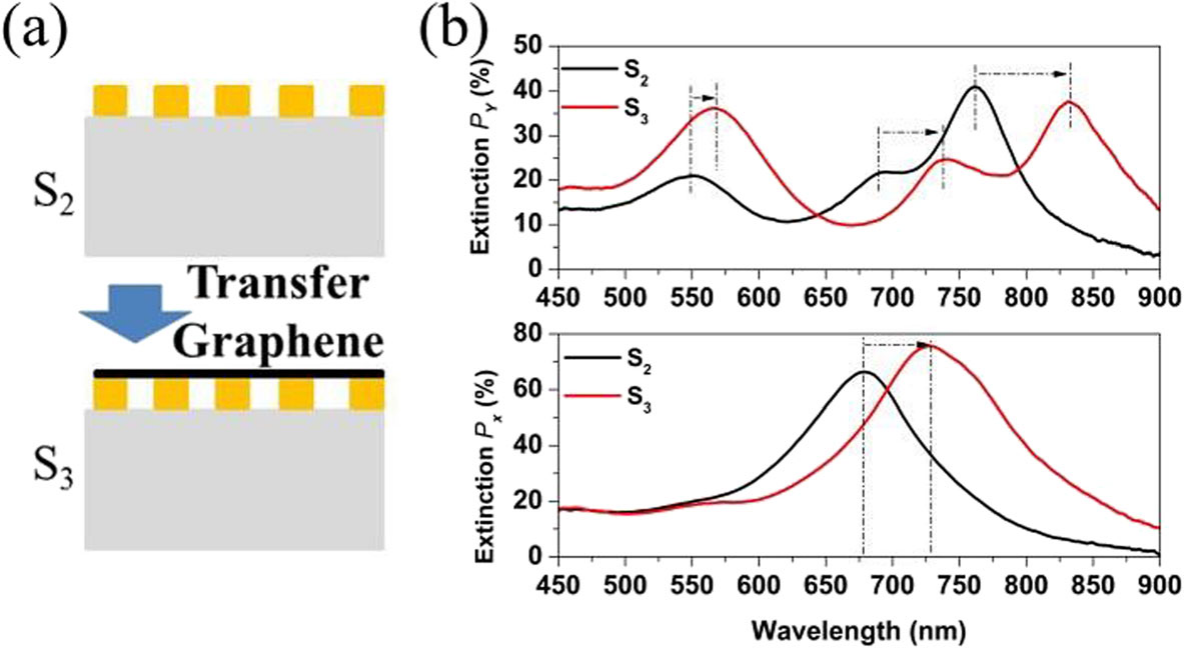
**a** Schematic illustration of transferring graphene above the Au plasmon resonator array. **b** The extinction spectra of the Au plasmon resonator array before and after transferring graphene

To further confirm the effect of graphene on the spectra of the Au plasmon resonator array at visible and NIR frequencies, we transferred a single-layer CVD graphene onto O_2_ RIE-treated sample. The transferred graphene covers the top surface of the Au plasmon resonator array as shown in Fig. 4a. The sample thus obtained is referred to as S_3_. The black curve and red curve in Fig. 4b show the extinction spectra of the Au plasmon resonator array before and after transferring graphene. We can see the presence of graphene on Au plasmon resonator array induces the plasmon resonance peaks to shift to red end, which is the same as the sample with graphene below the Au plasmon resonator array, as shown in Fig. 3b. So we can conclude that no matter whether graphene is located blow or on the Au plasmon resonator array, it predominantly causes the plasmon resonance peaks to shift to red end at visible and NIR frequencies. This behavior is similar to that of depositing dielectric film on silver nanoparticle [24], suggesting graphene predominantly shows a dielectric-like behavior at visible and NIR wavelength.

**Fig. 5:**
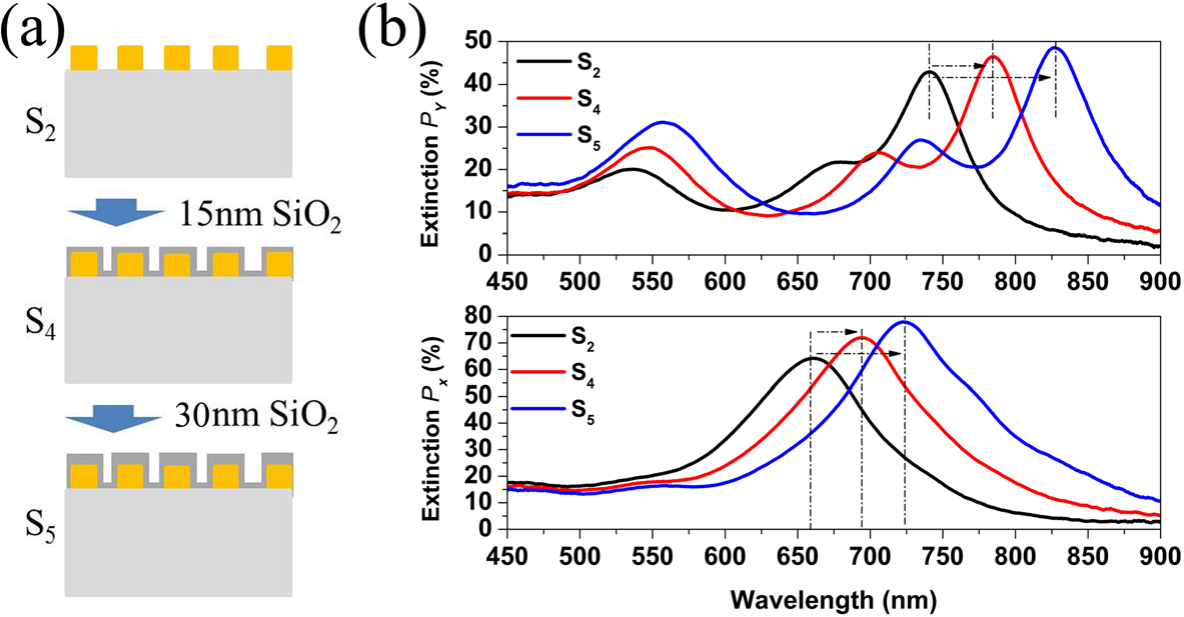
**a** Schematic illustration of E-beam evaporation SiO_2_ film onto the Au plasmon resonator array. **b** The extinction spectra of the Au plasmon resonator array with different thickness SiO_2_ film

To clearly show the behavior of Au plasmon resonator array after depositing dielectric film, we chose SiO_2_ as dielectric film and investigated the extinction spectra of Au plasmon resonator array after depositing different thickness SiO_2_ film on it. Figure 5a gives a brief illustration of the experimental process in which E-beam evaporates SiO_2_ film on the sample. We first removed the graphene on Au plasmon resonator array with RIE, then we used E-beam to evaporate 15 nm SiO_2_ film on the sample, and this process was repeated twice. In this way, sample S_4_ was covered with 15 nm SiO_2_ film and S_5_ with 30 nm. Figure 5b shows the extinction spectra of sample S_2_, S_4_, and S_5_. The extinction spectra of both S_4_ and S_5_ have a redshift for X- and Y-excitation as the thickness of SiO_2_ increases. For the samples excited by X-polarized light, plasmon resonance peak shows redshift from 660 to 695 nm when the thickness of SiO_2_ increased from 0 to 15 nm. When the thickness of SiO_2_ increased from 15 to 30 nm, the redshift of plasmon resonance peak increased further to 725 nm, as shown in bottom panel of Fig. 5b. The top panel in Fig. 5b shows the Y-polarized extinction peaks, which also exhibits a redshift as the SiO_2_ thickness increases. Their behaviors are similar no matter whether graphene is located on or below the Au plasmon resonator array. And furthermore, the redshift caused by a single layer graphene is equal to that brought about by 30 nm SiO_2_. Therefore, graphene predominantly shows dielectric-like behavior in Au plasmon resonator array at visible and NIR frequencies, and its effect is equal to about 30 nm SiO_2_.

The dielectric-like behavior of graphene at visible and NIR frequency can be attributed to the low carrier density in graphene compared with metals. In three dimension metal, the plasmon oscillation frequency at zero wave vector is

$$ {\omega}_{\mathrm{p},3\mathrm{D}}=\sqrt{\frac{4\pi {n}_{3\mathrm{D}}{e}^2}{m}}, $$

where *m* is the electron mass, *n*_*3D*_ is the free electron number per unit volume, and *e* is the electric charge. We get $$ {\upomega}_{\mathrm{p},3\mathrm{D}}=6\times {10}^{-4}{n_{3\mathrm{D}}}^{\raisebox{1ex}{$1$}\!\left/ \!\raisebox{-1ex}{$2$}\right.} $$. In two-dimensional graphene, the plasmon oscillation frequency in the long wavelength (*q* < <*k*_*F*_, where *q* is the wave vector, *k*_*F*_ is the Fermi vector) satisfy [25].

$$ {\omega}_{\mathrm{p},\mathrm{G}\mathrm{r}}=\sqrt{\frac{2q{e}^2{v}_{\mathrm{F}}}{\hslash \varepsilon}\sqrt{\pi {n}_{\mathrm{Gr}}}}, $$

where *q* is the wave vector, *v*_F_ = 1.1 × 10^6^ m/s is the Fermi velocity at SiO_2_ substrate [26], *ћ* is the reduced Plank constant, and *ε* is the dielectric constant. *n*_Gr_ is the free electron number per unit area. For a typical doping induced by SiO_2_ substrate [27], *n*_Gr_ ~ 10^11^/cm^2^, the plasmon oscillation frequency $$ {\upomega}_{\mathrm{p},\mathrm{G}\mathrm{r}}=7.5\times {10}^{-3}{n_{\mathrm{Gr}}}^{\raisebox{1ex}{$1$}\!\left/ \!\raisebox{-1ex}{$4$}\right.} $$ at *q* = 0.1k_F_. Furthermore, free electron in gold is about 6 × 10^22^/cm^3^, much higher than that in graphene. Therefore, *ω*_p*,*Gr_ *< <ω*_p*,*3D_ in the graphene and Au plasmon resonator system, and we can treat graphene as dielectric in engaging plasmon mode at visible and NIR frequency.

**Fig. 6:**
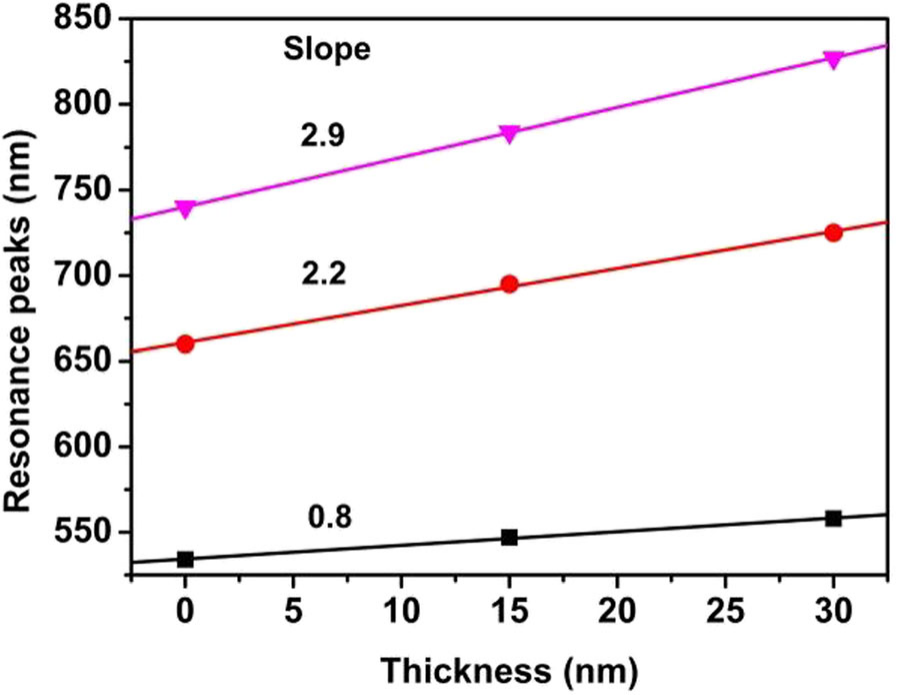
The evolution of the plasmon resonance peaks with the thickness of SiO_2_ film

Finally, we presented the dependence of plasmon resonance peaks on SiO_2_ film thickness in Fig. 6. The data shows the shift rate at red end is larger than that at blue end wavelength. For the resonance peak at about 535 nm, its shift rate is 0.8 nm/nm as SiO_2_ film increase. For the resonance peak at about 660 nm, its shift rate increases to 2.2 nm/nm. For the resonance peak at 725 nm, its shift rate further increases to 2.9 nm/nm. This discovery provides convenience to design wavelength tunable plasmon resonance and make more sensitive sensor based on plasmon resonance.

## Conclusions

In summary, we fabricated Au plasmon resonator array on quartz using graphene as discharge layer. The residual graphene on sample can be easily removed by O_2_ RIE, but we found the presence of graphene does not weaken and annihilate the plasmon resonance peaks, and it predominantly makes plasmon resonance peaks shift to the red end. Graphene shows dielectric-like behavior, which is similar to that of SiO_2_ film on Au plasmon resonator array, at visible and NIR frequencies. The research provides useful insight into the role of graphene in Au plasmon resonators and give some valuable guidance on designing graphene-based tunable plasmon resonators at visible and NIR frequency.

## Experimental Details

Graphene was grown by chemical vapor deposition (CVD) on a 25-μm thick copper (Cu) foil (Alfa Aesar, item No.13382) at 1000 °C using a mixture of methane (CH_4_) and hydrogen (H_2_). A protective polymethyl methacrylate (PMMA) film was applied to the deposited graphene layers, and the PMMA/graphene was transferred to the insulating substrate by dissolving the Cu foil using a 1 M FeCl_3_ solution. The transfer process was completed by attaching the PMMA/graphene to a quartz substrate followed by removal of the PMMA with acetone.

The process of EBL exposure and development on quartz substrates includes the following procedures. The quartz substrate coated with graphene was spin coated with resist PMMA 950 K A2 (MicroChem Company) at 3000 rpm, giving a resist thickness of 80 nm. The resist was prebaked on a hot plate at 180 °C for 90 s. The e-beam exposure was performed using a JEOL JBX-6300FS system, operated at 100 kV with beam current as 30 pA, beam diameter of 3 nm, exposure dose of 1100 μC/cm^2^, writing field of 62.5 μm, and field stitching error of about ±20 nm. The development was conducted in a 3:1 isopropyl alcohol (IPA) to methyl isobutyl ketone (MIBK) mixture for 90 s at room temperature followed by rinsing in IPA and drying by nitrogen flow. For the following process of pattern transfer, a metal layer was first deposited by an Anelva L-400EK e-beam evaporation system followed by a lift-off process using acetone at 40 °C. Graphene was removed using AVELVA reactive ion Etching (RIE) system, in which the O_2_ flow was calibrated in the following ways: flow rate was 40 sccm, pressure was 10 Pa, and RIE time was 15 s. SiO_2_ film was deposited using Anelva L-400EK e-beam evaporation system.

The optical spectra, including transmission and reflection, were measured using a Craic 308 PV microscopy spectrophotometer at room temperature and in the air.

## Additional files

Additional file 1: Figure S1. The absorption spectrum of single layer graphene. (JPG 132 kb)
Additional file 2: Figure S2. Confirmed by Raman spectrum. (JPG 191 kb)
