# Supplementary figures and images for: Dielectric-Like Behavior of Graphene in Au Plasmon Resonator
Source: Nanoscale Res Lett. 2016 Dec 7;11:541. doi: 10.1186/s11671-016-1753-6 (PMC5142172; doi:10.1186/s11671-016-1753-6)

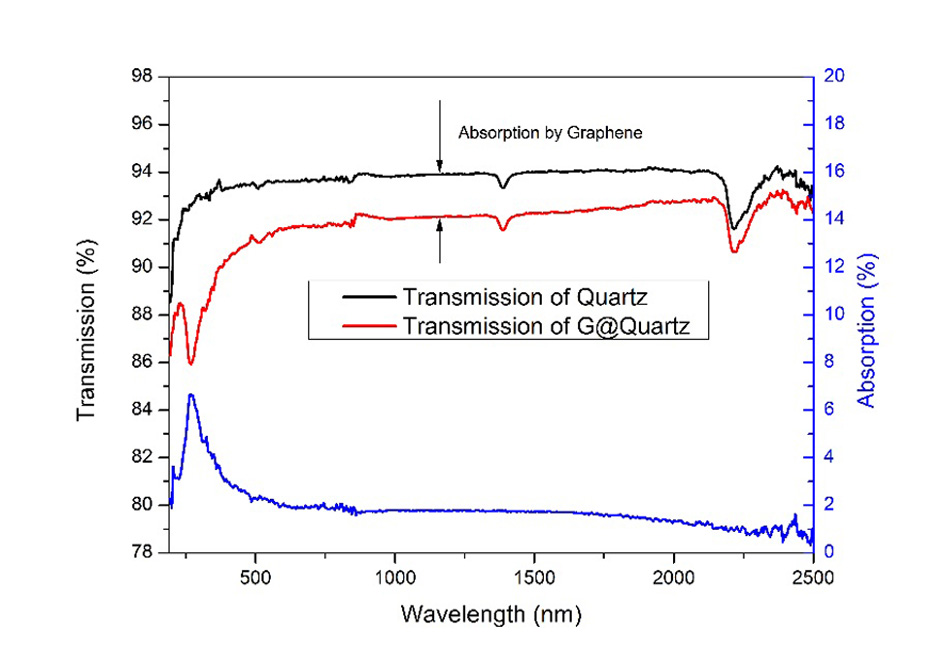

Supplement: Additional file 1: Figure S1. — The absorption spectrum of single layer graphene. (JPG 132 kb) [file 11671_2016_1753_MOESM1_ESM.jpg]

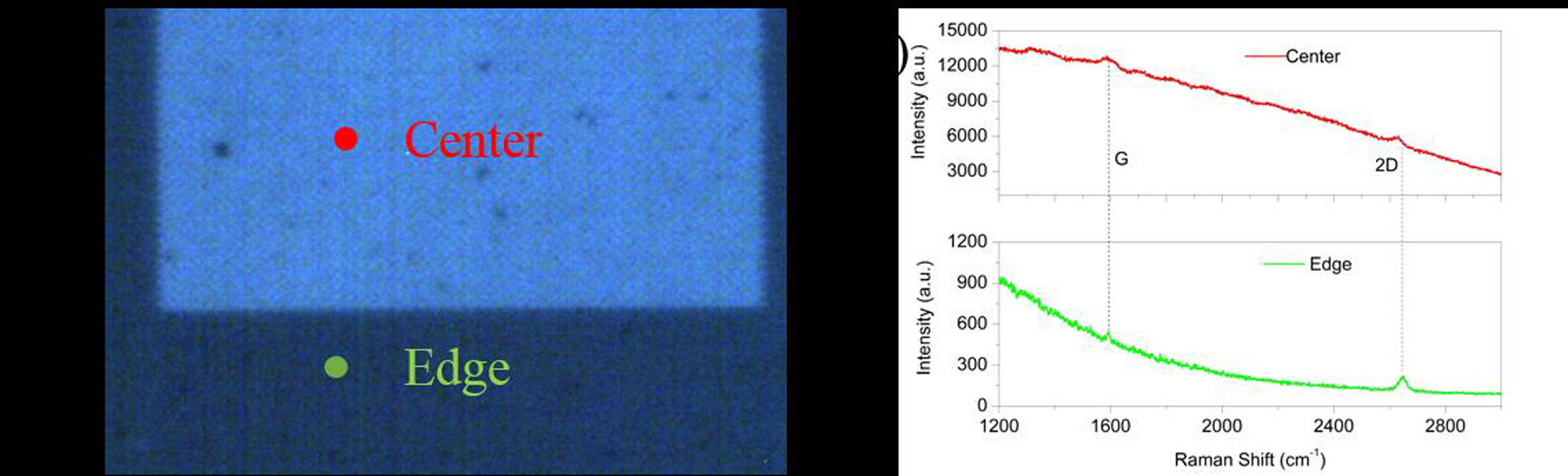

Supplement: Additional file 2: Figure S2. — Confirmed by Raman spectrum. (JPG 191 kb) [file 11671_2016_1753_MOESM2_ESM.jpg]
